# Parental Monitoring and Risk Behaviors and Experiences Among High School Students — Youth Risk Behavior Survey, United States, 2021

**DOI:** 10.15585/mmwr.su7201a5

**Published:** 2023-04-28

**Authors:** Patricia J. Dittus, Jingjing Li, Jorge V. Verlenden, Natalie J. Wilkins, Michelle N. Carman-McClanahan, Yolanda Cavalier, Melissa C. Mercado, Laura E. Welder, Douglas R. Roehler, Kathleen A. Ethier

**Affiliations:** ^1^Division of Adolescent and School Health, National Center for HIV, Viral Hepatitis, STD, and TB Prevention, CDC; ^2^Division of Violence Prevention, National Center for Injury Prevention and Control, CDC; ^3^Division of Injury Prevention, National Center for Injury Prevention and Control, CDC; ^4^Division of Overdose Prevention, National Center for Injury Prevention and Control, CDC

## Abstract

Parents have an important role in the promotion of healthy adolescent behaviors that can influence positive developmental trajectories and health outcomes. Parental monitoring is a central component of the parent-child relationship with the potential to reduce adolescent risk behaviors. Data from CDC’s 2021 nationally representative Youth Risk Behavior Survey were used to describe the prevalence of parental monitoring reported by U.S. high school students and examine associations between parental monitoring and adolescent behaviors and experiences. Behaviors and experiences included sexual behaviors, substance use, violence, and indicators of poor mental health. This report marks the first national assessment of parental monitoring among U.S. high school students. Point prevalence estimates and corresponding 95% CIs were generated in the bivariate analyses between parental monitoring and the outcomes, stratified by demographic characteristics (sex, race and ethnicity, sexual identity, and grade). Multivariable logistic regression analyses were conducted to estimate the main effects of parental monitoring (categorized as high = always or most of the time and low = never, rarely, or sometimes) for each outcome, controlling for all demographics. Overall, 86.4% of students reported that their parents or other adults in their family know where they are going or with whom they will be all or most of the time. Reports of high parental monitoring were protective for all risk behaviors and experiences, with models controlling for sex, race and ethnicity, sexual identity, and grade. Results highlight the need for public health professionals who develop public health interventions and programs to conduct further research on the relation between parental monitoring and student health outcomes.

## Introduction

Parents have an important role in the promotion of healthy adolescent behaviors that can influence developmental trajectories and health outcomes. Parental monitoring is an active, multidimensional process that includes setting boundaries and establishing an open exchange of information or knowledge related to a child’s whereabouts, companions, and activities (*1*). Parental monitoring has been identified as a central component of the parent-child relationship, with the potential to reduce risk behaviors, reduce involvement in situations that might involve high risk or negative behavior, and promote environmental contexts that support positive behavior and decision-making (*1*–*3*).

Previous studies have found protective associations between parental monitoring and multiple adolescent behaviors and experiences across race, ethnicity, and gender. For example, associations have been found between increased parental monitoring and adolescents’ decreased prevalence of ever having engaged in sexual intercourse as well as increased use of contraception or condoms if they do (*4*,*5*). Parental monitoring also has been associated with reduced intention to engage in risk behaviors such as drinking alcohol, using marijuana, and misusing prescription drugs (*3*,*6*). In addition, parental monitoring has been inversely associated with multiple violence-related outcomes, including reductions in bullying perpetration and victimization (e.g., school-based bullying, electronic or cyber-bullying), dating violence, and sexual violence (*7*,*8*). Less is known about the relations between parental monitoring and indicators of poor mental health. However, less parental monitoring has been associated with a greater likelihood of self-injury attempts, including suicide attempts (*9*,*10*). In addition, the role of parental monitoring in supporting adolescent health behaviors, reducing risk, and encouraging positive, healthy decision-making has not been studied comprehensively across adolescent subgroups. For example, additional research is needed to understand the protective role of parental monitoring for sexual minority adolescents.

One question assessing adolescents’ perceptions of parental knowledge of whereabouts and companions was included on the 2021 Youth Risk Behavior Survey (YRBS). This inclusion marks the first national, school-based assessment of student-reported parental monitoring and its association with adolescent behaviors and experiences. YRBS data from 2021 were used to both describe the prevalence of parental monitoring reported by youths and examine associations between high and low levels of parental monitoring and youth behaviors and experiences, including sexual behaviors, substance use, violence, and indicators of poor mental health. Findings from this report can be used to better understand the potential influence of parental monitoring and shape public health initiatives designed to improve adolescent health and well-being.

## Methods

### Data Source

This report includes data from the 2021 YRBS (N = 17,232), a cross-sectional, school-based survey conducted biennially since 1991. Each survey year, CDC collects data from a nationally representative sample of public and private school students in grades 9–12 in the 50 U.S. states and the District of Columbia. Additional information about YRBS sampling, data collection, response rates, and processing is available in the overview report of this supplement (*11*). The prevalence estimates for parental monitoring for the overall study population and by sex, race and ethnicity, grade, and sexual identity are available at https://nccd.cdc.gov/youthonline/App/Default.aspx. The full YRBS questionnaire, data sets, and documentation are available at https://www.cdc.gov/healthyyouth/data/yrbs/index.htm. This activity was reviewed by CDC and was conducted consistent with applicable federal law and CDC policy.*

### Measures

The main exposure of interest, parental monitoring, is derived from the question, “How often do your parents or other adults in your family know where you are going or with whom you will be?” Responses were combined to create two categories: high parental monitoring (always or most of the time) and low parental monitoring (never, rarely, or sometimes). The nine student health behaviors and experiences included sexual behaviors, substance use, violence, and mental health and suicide-related behaviors. Outcome variables were dichotomized (Table 1). Demographic variables included race and ethnicity (American Indian or Alaska Native [AI/AN], Asian, Black or African American [Black], Native Hawaiian or other Pacific Islander, White, Hispanic or Latino [Hispanic], and multiracial), sex (female or male), sexual identity (heterosexual, lesbian, gay, bisexual, questioning, or other), and grade (9 and 10 or 11 and 12). (Persons of Hispanic origin might be of any race but are categorized as Hispanic; all racial groups are non-Hispanic.) 

**TABLE 1:** Questions, response options, and denominators for health behaviors and experiences, by variable assessed — Youth Risk Behavior Survey, United States, 2021*

Variable	Question	Response options (analytic coding)	Denominator^†^
Parental monitoring	How often do your parents or other adults in your family know where you are going or with whom you will be?	Never, rarely, sometimes, most of the time, always (high = most of the time, always versus low = never, rarely, sometimes)	9,092
Ever had sex	Have you ever had sexual intercourse?	Yes, no (yes versus no)	12,157
Condom use^§^	The last time you had sexual intercourse, did you or your partner use a condom?	Yes, no (yes versus no)	3,314
Multiple partners	During your life, with how many persons have you had sexual intercourse?	I have never had sexual intercourse, 1 person, 2 persons, 3 persons, 4 persons, 5 persons, ≥6 persons (yes = ≥4 versus no = <4)	15,456
Current prescription opioid misuse	During the past 30 days, how many times did you take prescription pain medicine without a doctor’s prescription or differently than how a doctor told you to use it? (The lead-in for this question indicates “drugs such as codeine, Vicodin, OxyContin, Hydrocodone, and Percocet”.)	0 times, 1 or 2 times, 3–9 times, 10–19 times, 20–39 times, ≥40 times (yes = ≥1 versus no = 0)	9,866
Current marijuana use	During the past 30 days, how many times did you use marijuana?	0 times, 1 or 2 times, 3–9 times, 10–19 times, 20–39 times, ≥40 times (yes = ≥1 versus no = 0)	16,897
Forced sex	Have you ever been physically forced to have sexual intercourse when you did not want to?	Yes, no (yes versus no)	14,158
Electronic bullying	During the past 12 months, have you ever been electronically bullied?	Yes, no (yes versus no)	17,032
Persistent feelings of sadness or hopelessness	During the past 12 months, did you ever feel so sad or hopeless almost every day for 2 weeks or more in a row that you stopped doing some usual activities?	Yes, no (yes versus no)	16,961
Attempted suicide	During the past 12 months, how many times did you actually attempt suicide?	0 times, 1 time, 2 or 3 times, 4 or 5 times, ≥6 times (yes = ≥1 versus no = 0)	15,573

* N = 17,232 respondents.

^†^ The denominators are analytic sample sizes (unweighted).

^§^ Among sexually active youths.

### Analysis

Point prevalence estimates and corresponding 95% CIs were generated in the bivariate analyses between parental monitoring and the outcomes, stratified by demographic characteristics. Multivariable logistic regression analyses were conducted to estimate the main effects of parental monitoring for each outcome, controlling for all demographic characteristics. Estimates were considered statistically significant if the 95% CIs did not include 1.0 or if p was <0.05. Prevalence estimates with a denominator <30 were considered statistically unreliable and therefore were suppressed (*11*). All analyses were conducted in SAS-callable SUDAAN (version 11.0.3; RTI International) using sample weights to account for complex survey design and nonresponse.

## Results

Overall, 86.4% of students reported that their parents or other adults in their family know where they are going or with whom they will be all or most of the time (Table 2). High parental monitoring was more prevalent among females compared with males (89.3% versus 84.1%), more prevalent among Asian students compared with Black students (91.3% versus 79.8%), and less prevalent among students who self-identify as lesbian, gay, or bisexual compared with heterosexual or questioning or other students (84.2% versus 87.6% and 88.9%, respectively). By grade, no differences occurred in reports of parental monitoring.

**TABLE 2:** Prevalence of high school students who reported high levels of parental monitoring, by demographic characteristics — Youth Risk Behavior Survey, United States, 2021*

Characteristic	High parental monitoring^†^ % (95% CI)	p value^§^
**Overall**	86.4 (84.9–87.8)	NA
**Sex**		**<0.001**
Female	89.3 (87.9–90.6)	
Male	84.1 (81.9–86.0)	
**Race and ethnicity^¶^**		**<0.001**
American Indian or Alaska Native	84.4 (72.5–91.8)	
Asian**^,††^	91.3 (88.3–93.6)	
Black or African American^§§^	79.8 (75.5–83.5)	
Native Hawaiian or other Pacific Islander**^,††,§§,¶¶,^***^,†††^	—^§§§^	
White	88.5 (87.0–89.9)	
Hispanic or Latino**^,§§^	84.3 (81.9–86.4)	
Multiracial**	86.8 (81.9–90.6)	
**Sexual identity**		**0.013**
Heterosexual	87.8 (86.3–89.1)	
Lesbian, gay, or bisexual	84.2 (82.4–85.8)	
Questioning or other	88.9 (86.0–91.3)	
**Grade**		**0.057**
9 and 10	87.8 (85.4–89.8)	
11 and 12	85.2 (83.4–86.9)	

* N = 17,232 respondents. Because the state and local questionnaires differ by jurisdiction, students in these schools were not asked all national YRBS questions. Therefore, the total number (N) of students answering each question varied. Percentages in each category are calculated on the known data.

^†^ High parental monitoring is defined by a response of “most of the time, always” to the question, “How often do your parents or other adults in your family know where you are going or with whom you will be?”

^§^ p value is based on chi-square tests (p<0.05).

^¶^ Persons of Hispanic or Latino (Hispanic) origin might be of any race but are categorized as Hispanic; all racial groups are non-Hispanic.

** Significantly different from Black or African American students, on the basis of *t*-test analysis with Taylor series linearization (p<0.05).

^††^ Significantly different from Hispanic students, on the basis of *t*-test analysis with Taylor series linearization (p<0.05).

^§§^ Significantly different from White students, on the basis of *t*-test analysis with Taylor series linearization (p<0.05).

^¶¶^ Significantly different from Asian students, on the basis of *t*-test analysis with Taylor series linearization (p<0.05).

*** Significantly different from American Indian or Alaska Native students, on the basis of *t*-test analysis with Taylor series linearization (p<0.05).

^†††^ Significantly different from multiracial students, on the basis of *t*-test analysis with Taylor series linearization (p<0.05).

^§§§ ^Dash indicates cell data are suppressed because the denominator is <30 and therefore considered to be statistically unreliable.

The prevalences of nine health risk behaviors and experiences, stratified by level of parental monitoring and demographic characteristics, were calculated (Tables 3 and 4). Differences occurred in the prevalence of each outcome by sex, race and ethnicity, sexual identity, and grade. Compared with students who reported low levels of parental monitoring, students who reported high parental monitoring experienced more positive health outcomes (e.g., fewer sexual risk behaviors, less substance use, fewer experiences of violence, fewer mental health challenges, and fewer suicide attempts) and engaged in more protective behaviors (e.g., condom use). This pattern was particularly pronounced for lesbian, gay, or bisexual students with high parental monitoring.

**TABLE 3:** Prevalence of sexual behaviors and substance use behaviors among high school students, by demographic characteristics and levels of parental monitoring — Youth Risk Behavior Survey, United States, 2021*

Characteristic	Ever had sex^†^		Used condom^†^		Multiple partners^†^		Current prescription opioid misuse^†^		Current marijuana use^†^	
	High parental monitoring	Low parental monitoring	High parental monitoring	Low parental monitoring	High parental monitoring	Low parental monitoring	High parental monitoring	Low parental monitoring	High parental monitoring	Low parental monitoring
	% (95% CI)	% (95% CI)	% (95% CI)	% (95% CI)	% (95% CI)	% (95% CI)	% (95% CI)	% (95% CI)	% (95% CI)	% (95% CI)
**Sex**										
Female	28.7 (25.7–32.0)	54.5 (49.9–59.1)	48.4 (44.4–52.5)	37.7 (27.2–49.6)	4.3 (3.4–5.5)	12.8 (10.1–16.1)	6.8 (5.8–7.9)	18.7 (14.2–24.2)	15.7 (13.6–18.0)	39.6 (32.2–47.5)
Male	26.5 (24.2–28.9)	52.7 (47.5–57.7)	60.9 (55.1–66.3)	43.6 (35.1–52.5)	4.9 (4.1–5.9)	20.0 (16.0–24.7)	3.0 (2.4–3.8)	9.6 (6.5–13.3)	11.2 (9.5–13.1)	32.3 (27.4–37.5)
**Race and ethnicity^§^**										
American Indian or Alaska Native	27.2 (15.8–42.6)	—^¶^	—	—	7.8 (2.9–19.4)	—	4.5 (1.1–16.3)	—	13.8 (7.1–25.0)	—
Asian	10.4 (7.8–13.6)	25.1 (17.5–34.5)	—	—	2.0 (1.2–3.2)	3.0 (0.5–15.7)	3.3 (2.3–4.6)	12.7 (5.1–28.1)	4.4 (2.9–6.5)	16.9 (8.2–31.6)
Black or African American	34.5 (28.1–41.4)	56.5 (47.3–65.4)	50.6 (43.6–57.6)	44.0 (30.2–58.9)	7.0 (4.1–11.9)	23.5 (17.3–31.0)	7.3 (4.6–11.2)	14.2 (9.9–20.1)	18.7 (15.5–22.5)	35.7 (27.8–44.4)
Hispanic or Latino	29.1 (26.9–31.5)	53.6 (44.5–62.4)	50.0 (43.5–56.5)	45.8 (35.5–56.5)	5.4 (4.8–6.1)	14.0 (9.6–20.1)	6.9 (5.4–8.8)	16.8 (11.6–23.7)	14.8 (12.4–17.5)	34.7 (28.8–41.0)
Multiple races	32.8 (26.6–39.6)	44.8 (31.2–59.2)	—	—	5.3 (3.0–8.9)	13.9 (7.2–24.9)	4.6 (2.5–8.1)	12.4 (5.7–25.1)	18.6 (12.9–26.1)	40.6 (26.7–56.2)
White	27.5 (25.4–29.8)	55.5 (50.6–60.4)	57.4 (54.5–60.3)	38.0 (30.0–46.8)	4.1 (3.1–5.5)	18.3 (14.4–23.0)	3.8 (3.1–4.6)	10.1 (6.5–15.4)	12.5 (10.6–14.6)	36.7 (30.8–42.9)
**Sexual identity**										
Heterosexual	27.2 (24.8–29.6)	52.4 (48.2–56.7)	56.6 (53.4–59.9)	44.6 (37.8–51.6)	4.5 (3.9–5.3)	15.9 (13.3–18.9)	3.5 (3.0–4.1)	10.0 (7.0–14.0)	11.8 (10.3–13.4)	34.1 (31.0–37.3)
Lesbian, gay, or bisexual	35.9 (32.9–39.0)	60.7 (52.7–68.1)	39.4 (33.3–45.7)	38.7 (23.0–57.2)	5.7 (4.5–7.3)	19.5 (12.7–28.8)	9.5 (7.4–12.2)	21.7 (14.0–31.9)	23.3 (19.6–27.3)	43.9 (34.0–54.4)
Questioning or other	20.7 (17.0–24.9)	54.3 (43.9–64.4)	53.9 (43.1–64.4)	15.9 (6.8–32.6)	4.1 (2.3–7.3)	23.6 (14.7–35.7)	9.1 (6.9–11.9)	24.0 (14.3–37.6)	14.1 (10.5–18.6)	43.7 (29.7–58.8)
**Grade**										
9 and 10	17.2 (15.3–19.3)	41.8 (37.2–46.5)	58.6 (51.8–65.1)	49.9 (41.0–58.9)	2.3 (1.8–3.0)	10.1 (7.5–13.4)	5.2 (4.4–6.2)	12.2 (8.5–17.1)	9.5 (8.0–11.3)	26.3 (21.3–32.0)
11 and 12	38.5 (35.2–41.8)	62.1 (57.9–66.1)	52.3 (48.4–56.1)	37.5 (31.6–43.9)	7.2 (5.9–9.7)	22.4 (19.1–26.0)	4.6 (3.7–5.6)	13.3 (10.8–16.2)	17.5 (15.3–19.9)	41.6 (38.0–45.4)

* N = 17,232 respondents. Because the state and local questionnaires differ by jurisdiction, students in these schools were not asked all national YRBS questions. Therefore, the total number (N) of students answering each question varied. Percentages in each category are calculated on the known data.

^†^ Refer to Table 1 for variable definitions.

^§ ^Persons of Hispanic or Latino (Hispanic) origin might be of any race but are categorized as Hispanic; all racial groups are non-Hispanic.

^¶^ Dashes indicate cell data are suppressed because the denominator is <30 and therefore considered to be statistically unreliable.

**TABLE 4:** Prevalence of violence experiences, feeling sad and hopeless, and suicide attempts among high school students, by demographic characteristics and levels of parental monitoring — Youth Risk Behavior Survey, United States, 2021*

Characteristic	Forced sex^†^		Electronic bullying^†^		Sad and hopeless^†^		Suicide attempts^†^	
	High parental monitoring	Low parental monitoring	High parental monitoring	Low parental monitoring	High parental monitoring	Low parental monitoring	High parental monitoring	Low parental monitoring
	% (95% CI)	% (95% CI)	% (95% CI)	% (95% CI)	% (95% CI)	% (95% CI)	% (95% CI)	% (95% CI)
**Sex**								
Female	11.9 (10.3–13.7)	31.2 (26.5–36.4)	18.7 (17.1–20.5)	34.5 (30.8–38.3)	56.0 (53.2–58.8)	73.6 (68.8–77.9)	11.2 (9.7–12.9)	29.9 (25.2–34.9)
Male	2.6 (2.0–3.3)	8.1 (5.4–11.8)	10.5 (9.3–12.0)	16.8 (14.4–19.5)	26.9 (25.1–28.8)	45.1 (41.2–49.0)	4.5 (3.6–5.8)	13.7 (10.8–17.4)
**Race and ethnicity^§^**								
American Indian or Alaska Native	20.2 (11.8–32.2)	—^¶^	29.7 (18.4–44.2)	—	43.3 (31.3–56.2)	—	11.8 (6.1–21.6)	—
Asian	3.6 (2.4–5.4)	12.4 (6.2–23.1)	12.1 (9.1–15.9)	19.0 (11.9–28.8)	33.0 (28.6–37.7)	50.1 (24.7–75.4)	5.1 (2.9–8.6)	18.3 (3.5–32.4)
Black or African American	5.8 (4.3–7.8)	10.7 (7.7–14.8)	9.1 (7.5–11.0)	12.8 (9.0–17.8)	41.0 (36.7–45.5)	42.1 (35.3–49.2)	11.2 (8.4–14.9)	21.7 (14.6–31.1)
Native Hawaiian or other Pacific Islander	—	—	—	—	—	—	—	—
White	7.2 (6.3–8.4)	18.6 (13.9–24.4)	17.0 (14.8–19.4)	31.0 (26.2–36.3)	39.4 (37.0–41.9)	59.4 (54.0–64.5)	7.1 (5.9–8.5)	19.0 (13.8–25.5)
Hispanic or Latino	8.3 (6.9–10.0)	18.4 (12.8–25.6)	12.5 (9.2–16.7)	17.1 (13.0–22.2)	46.4 (43.7–49.2)	58.7 (51.3–65.6)	9.5 (7.9–11.3)	20.7 (16.3–25.8)
Multiracial	10.6 (7.9–14.0)	24.0 (15.6–35.2)	14.3 (9.2–21.4)	40.9 (28.0–55.0)	51.4 (46.3–56.4)	66.1 (51.4–78.1)	10.0 (7.4–13.4)	27.1 (14.8–44.4)
**Sexual identity**								
Heterosexual	4.3 (3.5–5.2)	9.9 (7.6–12.8)	12.1 (10.9–13.3)	17.9 (15.9–20.0)	33.5 (31.3–35.7)	51.6 (46.9–56.4)	4.8 (3.9–5.7)	13.6 (11.2–16.3)
Lesbian, gay, or bisexual	18.9 (16.4–21.7)	38.4 (30.1–47.4)	25.0 (21.4–29.0)	36.1 (28.8–44.1)	70.2 (66.2–73.8)	75.3 (67.1–81.9)	21.2 (17.7–25.1)	38.6 (28.9–49.3)
Questioning or other	13.9 (10.5–18.1)	38.9 (28.7–50.1)	22.4 (18.2–27.2)	54.2 (44.0–64.1)	66.4 (61.5–71.0)	79.8 (67.0–88.4)	13.1 (10.3–16.5)	46.8 (32.1–62.1)
**Grade**								
9 and 10	6.8 (5.7–8.2)	15.5 (11.5–20.5)	15.9 (14.4–17.5)	24.7 (20.4–29.7)	39.7 (37.4–42.1)	57.7 (51.9–63.3)	9.5 (8.3–10.8)	21.7 (16.8–27.6)
11 and 12	7.7 (6.5–9.2)	18.3 (15.5–21.6)	13.5 (12.1–15.0)	22.9 (19.2–27.1)	43.5 (41.5–45.5)	54.6 (51.2–58.0)	6.6 (5.7–7.6)	18.7 (14.4–23.9)

* N = 17,232 respondents. Because the state and local questionnaires differ by jurisdiction, students in these schools were not asked all national YRBS questions. Therefore, the total number (N) of students answering each question varied. Percentages in each category are calculated on the known data.

^†^ Refer to Table 1 for variable definitions.

^§^ Persons of Hispanic or Latino (Hispanic) origin might be of any race but are categorized as Hispanic; all racial groups are non-Hispanic.

^¶^ Dashes indicate cell data suppressed because the denominator is <30 and therefore considered to be statistically unreliable.

In the multivariable logistic regression analyses, reports of high parental monitoring were protective for all risk behaviors and experiences, with models controlling for sex, race and ethnicity, sexual identity, and grade (Table 5). For instance, prevalence of ever having had sex among high school students who reported high levels of parental monitoring was 54% lower compared with those who reported low levels of parental monitoring. Compared with low levels of parental monitoring, high levels of parental monitoring were associated with higher prevalence of using a condom at last sex and lower prevalence of reporting multiple lifetime sex partners. Similarly, high levels of parental monitoring were associated with lower prevalence of both current prescription opioid misuse and current marijuana use. In regard to experiences of violence, students who reported high levels of parental monitoring were less likely to have experienced forced sex in their lifetime and electronic bullying during the past 12 months than students who reported low levels of parental monitoring. Finally, high school students who reported high levels of parental monitoring were less likely to report persistent feelings of sadness and hopelessness and to have attempted suicide in the past 12 months than students who reported low levels of monitoring.

**TABLE 5:** Associations between parental monitoring and selected risk behaviors and experiences among high school students, by demographic characteristics — Youth Risk Behavior Survey, United States, 2021*

Characteristic	Ever had sex^†^	Condom use^†^	Multiple partners^†^	Current prescription opioid misuse^†^	Current marijuana use^†^	Forced sex^†^	Electronic bullying^†^	Sad and hopeless^†^	Suicide attempts^†^
	aPR (95% CI)	aPR (95% CI)	aPR (95% CI)	aPR (95% CI)	aPR (95% CI)	aPR (95% CI)	aPR (95% CI)	aPR (95% CI)	aPR (95% CI)
**Sex**									
Female	1.04 (0.95–1.15)	0.86 (0.74–1.00)^§,¶^	0.73 (0.60–0.90)^§^	1.72 (1.38–2.16)^§^	1.15 (1.05–1.25)^§^	3.26 (2.83–3.76)^§^	1.52 (1.32–1.76)^§^	1.69 (1.54–1.87)^§^	1.58 (1.27–1.97)^§^
Male	Ref	Ref	Ref	Ref	Ref	Ref	Ref	Ref	Ref
Race and ethnicity**									
American Indian or Alaska Native	0.92 (0.63–1.34)	0.76 (0.39–1.51)	1.75 (0.87–3.50)	1.03 (0.28–3.79)	0.96 (0.56–1.64)	2.34 (1.50–3.65)^§^	1.53 (1.00–2.35)^§^	1.13 (0.89–1.45)	2.22 (1.43–3.44)^§^
Asian	0.41 (0.33–0.50)^§^	1.12 (0.85–1.46)	0.34 (0.21–0.56)^§^	0.86 (0.55–1.33)	0.37 (0.24–0.58)^§^	0.53 (0.41–0.69)^§^	0.66 (0.48–0.93)^§^	0.81 (0.73–0.90)^§^	0.76 (0.46–1.24)
Black or African American	1.22 (1.04–1.43)^§^	0.90 (0.80–1.02)	1.61 (0.99–2.62)	1.66 (1.07–2.58)^§^	1.29 (1.07–1.54)^§^	0.72 (0.56–0.93)^§^	0.48 (0.33–0.58)^§^	0.97 (0.87–1.07)	1.42 (1.05–1.91)^§^
Native Hawaiian or other Pacific Islander	0.69 (0.42–1.14)	0.86 (0.16–4.63)	8.81 (6.60–11.76)^§^	1.88 (0.65–5.49)	0.78 (0.17–8.61)	1.21 (0.49–2.99)	0.48 (0.10–2.33)	0.97 (0.71–1.32)	1.04 (0.39–2.79)
White	Ref	Ref	Ref	Ref	Ref	Ref	Ref	Ref	Ref
Hispanic or Latino	1.06 (0.93–1.21)	0.89 (0.76–1.05)	1.15 (0.92–1.43)	1.64 (1.24–2.18)^§^	1.10 (0.92–1.30)	1.14 (0.97–1.34)	0.65 (0.49–0.87)^§^	1.13 (1.06–1.21)^§^	1.21 (0.99–1.47)
Multiracial	1.10 (0.91–1.32)	0.78 (0.62–0.97)^§^	1.05 (0.63–1.72)	1.00 (0.64–1.54)	1.29 (0.93–1.78)	1.20 (0.86–1.66)	0.81 (0.57–1.15)	1.17 (1.07–1.29)^§^	1.22 (0.91–1.62)
**Sexual identity**									
Heterosexual	Ref	Ref	Ref	Ref	Ref	Ref	Ref	Ref	Ref
Lesbian, gay, or bisexual	1.28 (1.17–1.40)^§^	0.76 (0.64–0.90)^§^	1.47 (1.14–1.90)^§^	2.07 (1.69–2.53)^§^	1.67 (1.45–1.93)^§^	2.82 (2.27–3.49)^§^	1.75 (1.46–2.10)^§^	1.70 (1.56–1.85)^§^	3.22 (2.52–4.12)^§^
Questioning or other	0.82 (0.72–0.94)^§^	0.87 (0.71–1.06)	1.18 (0.78–1.81)	2.17 (1.56–3.02)^§^	1.17 (1.01–1.34)^§^	2.31 (1.79–2.99)^§^	1.71 (1.43–2.05)^§^	1.60 (1.46–1.75)^§^	2.34 (1.94–2.94)^§^
**Grade**									
9 and 10	Ref	Ref	Ref	Ref	Ref	Ref	Ref	Ref	Ref
11 and 12	2.05 (1.88–2.23)^§^	0.83 (0.72–0.96)^§^	2.73 (2.23–3.33)^§^	0.95 (0.74–1.21)	1.73 (1.54–1.94)^§^	1.20 (1.04–1.38)^§^	0.86 (0.78–0.95)^§^	1.07 (1.02–1.13)^§^	0.74 (0.62–0.89)^§^
**Parental monitoring**									
High	0.54 (0.50–0.57)^§^	1.32 (1.15–1.52)^§^	0.31 (0.27–0.35)^§^	0.37 (0.31–0.45)^§^	0.39 (0.34–0.45)^§^	0.40 (0.31–0.51)^§^	0.58 (0.52–0.64)^§^	0.70 (0.66–0.74)^§^	0.39 (0.32–0.47)^§^
Low	Ref	Ref	Ref	Ref	Ref	Ref	Ref	Ref	Ref

**Abbreviations:** aPR = adjusted prevalence ratio; Ref = referent group.

* N = 17,232 respondents. Because the state and local questionnaires differ by jurisdiction, students in these schools were not asked all national YRBS questions. Therefore, the total number (N) of students answering each question varied. Percentages in each category are calculated on the known data.

^†^ Refer to Table 1 for variable definitions.

^§^ Estimates were considered statistically significant if the 95% CIs did not include 1.0.

^¶^ The unrounded value of the upper CI is 0.99; p = 0.047.

^**^ Persons of Hispanic or Latino (Hispanic) origin might be of any race but are categorized as Hispanic; all racial groups are non-Hispanic.

## Discussion

This report provides the first national prevalence estimates of adolescents’ experience of parental monitoring among U.S. high school students. Analyses of data collected in fall 2021 estimated that most students reported high levels of parental monitoring, defined in this report as parent knowledge of where a student was going and with whom. Although differences occurred in experience of parental monitoring by sex, race and ethnicity, sexual identity, and grade, overall 86% of students across all groups said their parents knew where they were and with whom they would be.

Associations between levels of students’ experience of parental monitoring and behaviors and experiences that affect the health and well-being of adolescents, including sexual behaviors, substance use, violence, mental health, and suicide-related behaviors, also were examined. For all behaviors and experiences included in this report, high parental monitoring was associated with lower risk for negative outcomes. Of note, the measure of parental monitoring used in this report reflects students’ perceptions of whether their parents know where they are and with whom. This measure might indicate various interrelated factors, including parental behaviors (e.g., positive communication and inquiry) and adolescent disclosure, and might reflect positive parent-child relationships and family connectedness. Previous research has found that adolescents’ perceptions of parents’ knowledge of their whereabouts and companions are influenced by both solicitation of information by parents and relationship satisfaction reported by adolescents (*5*). The multidimensional nature of the construct indicates that it is related to a broad set of behaviors (i.e., activities in which adolescents engage, such as sex and substance use) and experiences (i.e., things that happen to adolescents). The multiple factors likely influencing whether students disclose their whereabouts and companions to their parents might be related in different ways to the outcomes of interest and might lead to different promotion strategies.

For instance, parental knowledge of students’ whereabouts can prevent opportunities for engaging in risk behaviors or for spending time with peers who might promote such behaviors (*1*,*3*,*6*). In this report, high parental monitoring was inversely related to student reports of ever having sex, multiple sex partners, and for male students, increased prevalence of condom use. These findings support previous research demonstrating that parental monitoring positively affects decisions about sexual activity among young persons (*4*–*7*). Similarly, observed relations between parental monitoring and decreased substance use in this report are congruent with analyses from the National Survey on Drug Use and Health, other longitudinal studies (*12*), and parenting interventions targeting adolescent substance use (*3*).

High parental monitoring also was related to lower prevalence of electronic bullying victimization and forced sex. Previous studies have found that collaborative parental monitoring strategies (e.g., those focused on communication) are associated with lower cyber-bullying victimization and perpetration, and family connectedness is associated with decreased experience of violence victimization and perpetration (*2*,*7*,*8*). Building strong relationships with parents and other prosocial adults might be an especially important protection for students at increased risk for violence (*7*,*8*). CDC’s youth violence and adverse childhood experiences (ACEs) technical packages provide examples of the best available evidence for the prevention of youth violence and ACEs, including parenting skills and family relationship programs that support caregivers and teach communication, problem-solving, and behavior monitoring and management skills (*13*,*14*).

In this report, a strong relation was found between students’ perceptions of parental monitoring and improved mental health and decreased suicidality. High parental monitoring was associated with lower likelihood of reporting symptoms of poor mental health, including feeling sad and hopeless and having attempted suicide. This finding adds to studies that have found a weak negative association between parental monitoring and depression (*9*). In another study, parental monitoring also was negatively correlated with suicidality, self-injury, and depression, such that increased monitoring was associated with decreased poor outcomes (*10*). The link between parental knowledge of companions and whereabouts and students’ mental health and suicidality is less direct. This link aligns, however, with other research on family relationships and connectedness (*15*), suggesting that monitoring knowledge expressed by students is likely the result of positive relationships rather than parental control of activities. In fact, parental monitoring strategies that facilitate involvement, information sharing, and parental warmth and support have demonstrated potential for reducing risks for poor mental health outcomes (https://www.cdc.gov/suicide/pdf/preventionresource.pdf).

Overall, parental monitoring had universal positive effects across all domains of risk behavior and experiences investigated in this report. Systematic reviews of parental monitoring literature have found similar protective associations between parental monitoring and youth risk behaviors, including substance use and risky sexual activity (*2*–*4*,*6*). However, among students with a history of social isolation and societal marginalization, including those who identify as lesbian, gay, bisexual, questioning, or other, effectiveness of parental monitoring has been tied to strategies that focus on the establishment of positive home environments and family relationships where students are comfortable disclosing information and feel accepted, rather than just focus on limiting opportunities for sexual activity (*16*). The findings discussed in this report warrant further exploration and research on specific aspects of parental monitoring and engagement that are most strongly tied to positive youth health behaviors and outcomes.

## Future Directions

Parental monitoring is a broad construct that encompasses a range of interrelated actions that include information exchange between parents and students. Measurements of parental monitoring vary, with certain measures attending more to parental actions and parental sense of control and others incorporating adolescents’ willingness to disclose information to parents (*1*). The student perspective of parental monitoring represented by the YRBS measure considers adolescent information sharing, representing student perceptions of parental knowledge. Further research is needed to assess measurement quality and explore the relation between other dimensions of parental monitoring and student health outcomes. Additional research also is needed to explore factors that might affect parental monitoring practices (e.g., neighborhood social cohesion, parent-adolescent relational quality, and cultural values) that might support increased parental monitoring and engagement. Such research is needed for the design of public health interventions and programming. Future work could explore protective qualities of parental monitoring across intersecting student and parent identities (e.g., race and ethnicity and sexual identity) and attributes of interventions to improve parental monitoring and adolescent outcomes.

## Limitations

General limitations for the YRBS are available in the overview report of this supplement (*11*). The findings in this report are subject to at least three additional limitations. First, causality between parental monitoring and student behaviors and experiences cannot be inferred by these cross-sectional data. Second, the single-item measure of perceived parental monitoring might not capture the complexity of this construct because parental monitoring knowledge might be gained through a combination of voluntary youth disclosure of information, parental solicitation of information, and parental control strategies such as rule enforcement (*17*). Finally, although the examples provided are only of opioid-containing prescription medications, the assessment of prescription opioid misuse might be overestimated because the questions refer to prescription pain medication more generally.

## Conclusion

Adolescents need support and guidance to promote healthy behavioral decisions and development. The nationally representative findings from the 2021 YRBS provide evidence of the potential effectiveness of parental monitoring in reducing adolescent risk behaviors, negative experiences, and subsequent outcomes. Understanding factors that influence effective parental monitoring and parenting practices that foster supportive relationships and home environments represent important next steps in this area of research.

## References

[1] Guilamo-Ramos V, Jaccard J, Dittus P. Parental monitoring of adolescents: current perspectives for researchers and practitioners. New York, NY: Columbia University Press; 2010.

[2] Elsaesser C, Russell B, Ohannessian CM, Patton D. Parenting in a digital age: a review of parents’ role in preventing adolescent cyberbullying. Aggress Violent Behav 2017;35:62–72. 10.1016/j.avb.2017.06.004

[3] Kuntsche S, Kuntsche E. Parent-based interventions for preventing or reducing adolescent substance use—a systematic literature review. Clin Psychol Rev 2016;45:89–101. 10.1016/j.cpr.2016.02.004 27111301

[4] Dittus PJ, Michael SL, Becasen JS, Gloppen KM, McCarthy K, Guilamo-Ramos V. Parental monitoring and its associations with adolescent sexual risk behavior: a meta-analysis. Pediatrics 2015;136:e1587–99. 10.1542/peds.2015-0305 26620067 PMC5513153

[5] Ethier KA, Harper CR, Hoo E, Dittus PJ. The longitudinal impact of perceptions of parental monitoring on adolescent initiation of sexual activity. J Adolesc Health 2016;59:570–6. 10.1016/j.jadohealth.2016.06.011 27567066 PMC6739880

[6] Ryan J, Roman NV, Okwany A. The effects of parental monitoring and communication on adolescent substance use and risky sexual activity: a systematic review. Open Fam Stud J 2015;7:7–12. 10.2174/1874922401507010012

[7] Doty JL, Gower AL, Rudi JH, McMorris BJ, Borowsky IW. Patterns of bullying and sexual harassment: connections with parents and teachers as direct protective factors. J Youth Adolesc 2017;46:2289–304. 10.1007/s10964-017-0698-0 28584921

[8] Khetarpal SK, Szoko N, Culyba AJ, Ragavan M. The role of parental monitoring as a protective factor against youth violence victimization. J Adolesc Health 2021;68(Suppl 2):S51–2. 10.1016/j.jadohealth.2020.12.106

[9] Yap MB, Jorm AF. Parental factors associated with childhood anxiety, depression, and internalizing problems: a systematic review and meta-analysis. J Affect Disord 2015;175:424–40. 10.1016/j.jad.2015.01.050 25679197

[10] MacPherson HA, Wolff J, Nestor B, Parental monitoring predicts depressive symptom and suicidal ideation outcomes in adolescents being treated for co-occurring substance use and psychiatric disorders. J Affect Disord 2021;284:190–8. 10.1016/j.jad.2021.02.021 33607509 PMC7926270

[11] Mpofu JJ, Underwood JM, Thornton JE, Overview and methods for the Youth Risk Behavior Surveillance System—United States, 2021. In: Youth Risk Behavior Surveillance—United States, 2021. MMWR Suppl 2023:72(No. Suppl 1):1–12. 37104281 10.15585/mmwr.su7201a1 PMC10156160

[12] Donaldson CD, Nakawaki B, Crano WD. Variations in parental monitoring and predictions of adolescent prescription opioid and stimulant misuse. Addict Behav 2015;45:14–21. 10.1016/j.addbeh.2015.01.022 25622102 PMC5902021

[13] David-Ferdon C, Vivolo-Kantor AM, Dahlberg LL, Marshall KJ, Rainford N, Hall JE. A comprehensive technical package for the prevention of youth violence and associated risk behaviors. Atlanta, GA: US Department of Health and Human Services, CDC, National Center for Injury Prevention and Control; 2016. https://www.cdc.gov/violenceprevention/pdf/yv-technicalpackage.pdf

[14] CDC. Preventing adverse childhood experiences: leveraging the best available evidence. Atlanta, GA: US Department of Health and Human Services, CDC, National Center for Injury Prevention and Control; 2019. https://www.cdc.gov/violenceprevention/pdf/preventingACES.pdf

[15] Steiner RJ, Sheremenko G, Lesesne C, Dittus PJ, Sieving RE, Ethier KA. Adolescent connectedness and adult health outcomes. Pediatrics 2019;144:1–11. 10.1542/peds.2018-3766 31235609 PMC9125410

[16] Thoma BC, Huebner DM. Parental monitoring, parent-adolescent communication about sex, and sexual risk among young men who have sex with men. AIDS Behav 2014;18:1604–14. 10.1007/s10461-014-0717-z 24549462 PMC4108561

[17] Stattin H, Kerr M. Parental monitoring: a reinterpretation. Child Dev 2000;71:1072–85. 10.1111/1467-8624.00210 11016567

